# Effects of starting one lung ventilation and applying individualized PEEP right after patients are placed in lateral decubitus position on intraoperative oxygenation for patients undergoing thoracoscopic pulmonary lobectomy: study protocol for a randomized controlled trial

**DOI:** 10.1186/s13063-024-08347-8

**Published:** 2024-07-22

**Authors:** Qing-Yuan Wang, Yang Zhou, Meng-Rui Wang, You-You Jiao

**Affiliations:** Department of Anesthesiology, Peking University Third Hospital, NO. 49, North Garden Road, Haidian District, Beijing, People’s Republic of China

**Keywords:** One lung ventilation, Individualized PEEP, Video-assisted thoracic surgery, Atelectasis, Recruitment maneuvers, Oxygenation

## Abstract

**Background:**

For patients receiving one lung ventilation in thoracic surgery, numerous studies have proved the superiority of lung protective ventilation of low tidal volume combined with recruitment maneuvers (RM) and individualized PEEP. However, RM may lead to overinflation which aggravates lung injury and intrapulmonary shunt. According to CT results, atelectasis usually forms in gravity dependent lung regions, regardless of body position. So, during anesthesia induction in supine position, atelectasis usually forms in the dorsal parts of lungs, however, when patients are turned into lateral decubitus position, collapsed lung tissue in the dorsal parts would reexpand, while atelectasis would slowly reappear in the lower flank of the lung. We hypothesize that applying sufficient PEEP without RM before the formation of atelectasis in the lower flank of the lung may beas effective to prevent atelectasis and thus improve oxygenation as applying PEEP with RM.

**Methods:**

A total of 84 patients scheduled for elective pulmonary lobe resection necessitating one lung ventilation will be recruited and randomized totwo parallel groups. For all patients, one lung ventilation is initiated the right after patients are turned into lateral decubitus position. For patients in the study group, individualized PEEP titration is started the moment one lung ventilation is started, while patients in the control group will receive a recruitment maneuver followed by individualized PEEP titration after initiation of one lung ventilation. The primary endpoint will be oxygenation index measured at T4. Secondary endpoints will include intrapulmonary shunt, respiratory mechanics, PPCs, and hemodynamic indicators.

**Discussion:**

Numerous previous studies compared the effects of individualized PEEP applied alone with that applied in combination with RM on oxygenation index, PPCs, intrapulmonary shunt and respiratory mechanics after atelectasis was formed in patients receiving one lung ventilation during thoracoscopic surgery. In this study, we will apply individualized PEEP before the formation of atelectasis while not performing RM in patients allocated to the study group, and then we’re going to observe its effects on the aspects mentioned above. The results of this trial will provide a ventilation strategy that may be conductive to improving intraoperative oxygenation and avoiding the detrimental effects of RM for patients receiving one lung ventilation.

**Trial registration:**

www.Chictr.org.cn ChiCTR2400080682. Registered on February 5, 2024.

**Supplementary Information:**

The online version contains supplementary material available at 10.1186/s13063-024-08347-8.

## Background

Nowadays, most video-assisted thoracic surgeries require one lung ventilation to avoid contamination of the contralateral lung by secretions and blood from the operated lung and to facilitate surgical exposure. Patients receiving one lung ventilation in lateral decubitus position are prone to develop atelectasis in the dependent lung due to decreased thoracic compliance and compression of the mediastinum and abdominal contents [1]. Increased atelectatic regions in the dependent lung not only give rise to lung injury, but also increase intrapulmonary shunt, which aggravates hypoxemia [2]. Therefore preventing formation of atelectasis during one lung ventilation has become a crucial problem.

Many randomized controlled trials discovered that a lung protective ventilation strategy involving low tidal volume combined with PEEP and RM could significantly reduce driving pressure, peak airway pressure, plateau pressure anddead space, alleviate intrapulmonary shunt, improve oxygenation and reduce the incidence of PPCs; whereas the ventilation mode that incorporated low tidal volume combined with PEEP but without RM had limited effects on oxygenation and incidence of PPCs [1, 3–7].

However, RM could cause delayed pulmonary inflammatory response and lung injury [1]. Furthermore, the high airway pressure required to reopen collapsed alveoli during RM could lead to overinflation of lung units, and thus could shift blood flow to atelectatic lung units, disrupt gas exchange, and induce inflammatory reaction [8].

Although RM have become an element of lung protective ventilation, no consensus has been reached concerning the airway pressure and the length of time needed to perform RM [9]. In the study of Miura and colleagues, in which response to RM was defined as increase in end expiratory lung volume equaling to or exceeding 20% of baseline level, half of the patients involved were responsive to RM. Compared to responders, the non-responders’ proportion of atelectatic lung tissue is much smaller, limiting the effect of RM on improving oxygenation and enlarging lung volume [10]. Consequently, the benefit of RM performed before formation of atelectasis was limited.

Among patients receiving general anesthesia with muscle relaxants, three in four would develop atelectasis [11]. According to CT results, atelectasis usually forms in gravity dependent areas no matter in which position patients are placed [11]. Therefore, in supine position, atelectasis usually forms in the dorsal part of the lung, while in lateral decubitus position, atelectasis tends to develop in the lower flank of the lung. Accordingly, after patients are switched from supine to lateral decubitus position, the collapsed lung units in the dorsal part of the lung will reexpand, while atelectasis will slowly develop in the lower flank of the lung. So it’s reasonable to believe that development of atelectasis could be prevented by applying sufficient PEEP before lung units in the lower flank start to collapse.

In many previous studies, patients received two lung ventilation without PEEP after being placed in lateral decubitus position, and one lung ventilation wasn’t initiated until pleura opening [3, 4, 6]. Immediately after initiation of one lung ventilation, RM were performed followed by individualized PEEP (iPEEP) titration. Because of the long interval between the initiation of two lung ventilation in lateral decubitus position and the initiation of one lung ventilation, during which period patients were ventilated with fraction of inspired oxygen (FiO_2_) of 100% and without PEEP, most patients had already developed atelectasis in the lower flank of the dependent lung and consequently necessitated RM to reopen collapsed alveoli.

The study of Edmark et al. which enrolled patients with body mass index (BMI) over 35 kg/m^2^ undergoing elective laparoscopic gastric volume reduction surgery discovered that patients who received 10cmH_2_O continuous positive airway pressure (CPAP) or PEEP during anesthesia induction and preoxygenation without RM exhibited preserved oxygenation throughout the entire duration of anesthesia until emergence [12]. Because obese patients under general anesthesia are more susceptible to developing atelectasis compared to their normal weight counterparts [13], we believe that atelectasis formation may be prevented throughout the whole duration of surgery by applying sufficient PEEP while not performing RM.

Numerous studies confirmed the advantages of iPEEP for patients undergoing one lung ventilation under general anesthesia [5, 12, 14, 15]. However, up till now, no consensus has been reached concerning the appropriate method for iPEEP titration. When tidal volume remains constant, driving pressure mainly correlates with Cstat; so PEEP could be titrated to maximize Cstat [15]. According to the result of a meta-analysis, the oxygenation of patients receiving driving-pressure-guided lung protective ventilation during one lung ventilation was significantly higher than those ventilated with other strategies [5]. Since increase in driving pressure strongly correlates with increased incidence of PPCs, we believe that for patients receiving one lung ventilation, titrating PEEP to optimize Cstat has its advantages.

This trial is going to test the hypothesis: the ventilation strategy of starting one lung ventilation and then iPEEP titration immediately after adjustment of the position of the double lumen tube in lateral decubitus position while not performing RM is not inferior to the strategy of initiating one lung ventilation right after pleura opening and then performing RM followed by iPEEP titration in the aspects of improving patients’ oxygenation, respiratory mechanics and reducing the incidence of PPCs during one lung ventilation.

## Methods/design

### Objectives and design

**Fig. 1:**
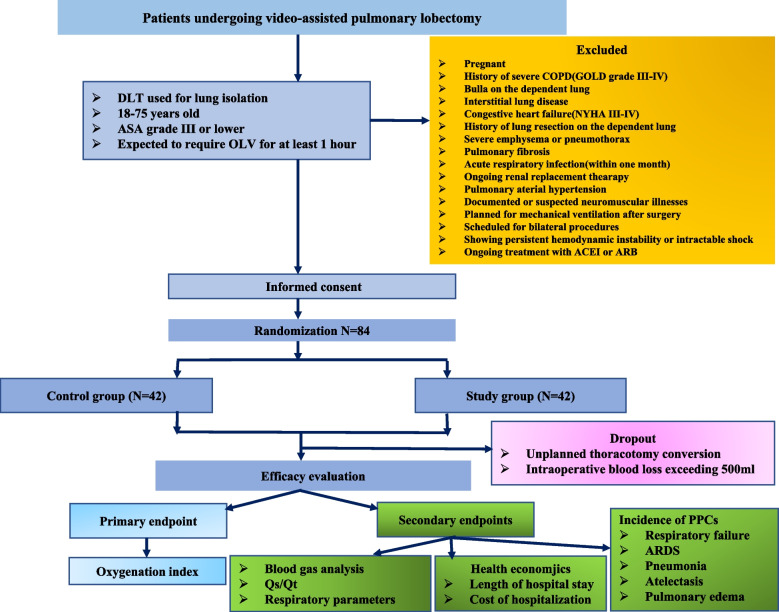
Consolidated standards of reporting trials (CONSORT) diagram of this trial. *DLT* double lumen tube, *COPD* chronic obstructive pulmonary disease, *ASA* American society of anesthesiologists, *OLV* one lung ventilation, *ARDS* acute respiratory distress syndrome, *Qs/Qt* fraction of intrapulmonary shunt

This parallel, two-arm, single-center, prospective, single blind (investigator-initiated, assessor-blinded), randomized, controlled trial tests the hypothesis that applying sufficient PEEP without RM right after patients are turned into lateral decubitus position is an effective strategy for patients receiving one lung ventilation. 84 patients will be randomly divided into one of two different groups (see Consolidated Standards of Reporting Trials [CONSORT] diagram, Fig. 1). The SPIRIT 2013 Checklist is given in Additional file 1.

This trial will be conducted at Peking University Third Hospital, Beijing, China. The study complies with the CIOMS Principles of the International Guidelines for Biomedical Research Involving Human Subjects and the WMA of the Declaration of Helsinki. The study has obtained ethics approval from the Ethics Committee of Peking University Third Hospital (The approval number is 2023–614-03) and has been registered in the Chinese Clinical Registry (Chictr) (registration number: ChiCTR2400080682).

### Blinding, data collection, randomization and record keeping

This is a single-blind randomized clinical trial. Demographic data, results of laboratory tests, history of previous illnesses, fluid balance, hemodynamic variables, anesthesia-associated variables, respiratory parameters and postoperative clinical status will be collected and recorded onto case report forms (CRF). All patients meeting the inclusion criteria will be randomly allocated to the study group and the control group in a ratio of 1:1. Randomization will be carried out by means of a computer-generated blocked randomization form, with 17 blocks of five patients per block. Allocation will proceed with opaque, sealed and numbered envelopes. Subjects will be recruited and assigned in numeric order. All original records (CRF and associated correspondence) will be archived and safeguarded for 10 years and then destroyed, as required by hospital standards.

### Data monitoring

A principal investigator and other members who take part in the design and implementation of the trial compose the research team. For the sake of quality control, a physician who does not take part in the trial will be in charge of data monitoring. Monitoring comprises evaluation of study progress and verification of precision as well as the integrity of recorded data. After completion of the study, the original data and results will be submitted to the scientific management committee, and will be disclosed to the public after publication of results.

### Auditing trial conduct

A physician independent from the investigators will be responsible for supervising the implementation of the trial and ensuring that the trial comply with the rigorous standards formulated by the state food and drug administration for good clinical practice. Trial conduct will be audited every twelve months across the trial timeline.

### Study population

Patients scheduled for elective video-assisted pulmonary lobe resection under general anesthesia will be screened and recruited during routine preoperative assessment. During recruitment, we will cooperate with surgeons to obtain detailed medical history and results of laboratory tests and radiological tests. Furthermore, we will discuss the procedures of our trial intervention with other anesthesiologists of our center, thereby receiving assistance from collegues during the trial implemention. With the help of collegues, reaching sample size wouldn’t be difficult. The inclusion criteria are as follows: 18–75 years old, American society of anesthesiologists (ASA) grade III or lower, double lumen tube used for lung isolation, length of one lung ventilation exceeding that of two lung ventilation, anticipated to undergo one lung ventilation for over an hour.

Exclusion criteria: pregnant, history of chronic obstructive pulmonary disease (COPD) (defined as GOLD grades III-IV), with pulmonary fibrosis, requiring devices other than double lumen tube for lung isolation, with bulla on the dependent lung, with severe pneumothorax or emphysema, with congestive heart failure (defined as NYHA grades III-IV), having a history of pulmonary surgery, a history of acute respiratory infection (within one month prior to surgery), dependent on renal replacement therapy, pulmonary arterial hypertension (defined as mean pulmonary arterial pressure exceeding 25 mmHg during rest or systolic pulmonary arterial pressure over 40 mmHg), known or suspected neuromuscular illnesses (thymoma, myasthenia gravis, myopathy, myodystrophy), planned for postoperative mechanical ventilation, scheduled for bilateral surgery, scheduled for surgery in prone position, sustained hemodynamic instability or refractory shock (according to the judgement by the anesthesiologist in charge), known significant lung injury (PaCO_2_ > 50 mmHg, PaO_2_ < 50 mmHg), taking angiotensin converting enzyme inhibitor (ACEI) or angiotensin receptor blocker (ARB) to treat hypertension.

Dropout criteria: unplanned thoracotomy conversion, intraoperative blood loss surpassing 500 ml.

### Anesthesia

An arterial catheter will be inserted into patients’ radial artery or dorsal pedis artery under local anesthesia without sedation. Intraoperative monitoring includes 5-lead electrocardiogram, invasive arterial blood pressure, pulse oximetry and train of four (TOF) stimulation. Anesthesia is induced with 1-2 mg/kg propofol, 0.2 mg/kg etomidate, 1–1.5 mg/kg lidocaine and 0.3ug/kg sufentanil and 0.15 mg/kg cisatracurium. After preoxygenation with pure oxygen, patients’ trachea is intubated with a double lumen tube of appropriate side and size. Sevoflurane and remifentanil are used for anesthesia maintainence throughout the entire surgical procedure. Inhaled concentration of sevoflurane are adjusted to maintaining bispectral index(BIS) value within the range of 40 to 60. 0.03 mg/kg cisatracurium is intravenously injected intermittently to keep TOF ratio within T1 to T3.

Lactate ringer’s solution is infused intraoperatively, the infusion rate of which is adjusted to sustaining urine output above 0.5 ml/kg/h. The range of intraoperative mean arterial pressure (MAP) fluctuation is held within 20% from baseline. Intraoperative heart rate is sustained within 50-100 bpm. Norepinephrine is infused continuously to avoid hypotension. A single dose of 4-8ug norepinephrine is injected when necessary. Esmolol is used to treat hypertension after intubation, while a single dose of 0.03 mg/kg nicardipine is injected to treat hypertension during emergence. Infusion of norepinephrine is slowed down or halted upon the occurrence of hypertension. If significant drop in blood pressure is not observed a few minutes later, a single dose of 0.1ug/kg sufentanil is infused or the infusion rate of remifentanil is elevated as an alternative. If hypertension is not attenuated after a few minutes, esmolol (in the case of tachycardia) or nicardipine is used. In the case of conjunction of bradycardia (heart rate < 50 bpm) and hypotension, a single dose of 5-10 mg ephedrine is injected. Anisodamine is injected when bradycardia occurs without the presence of hypotension. In the case of conjunction of tachycardia (heart rate > 100 bpm) and hypotension, the infusion rate of lactate ringer’s solution is elevated or a single dose of 40ug phenylephrine is injected.

Inhalation of sevoflurane is stopped when the surgery is coming to an end, while the infusion rate of remifentanil is maintained at 0.05ug/kg/min from the end of the surgery to extubation. If T4/T1 remains below 90% during emergence, neostigmine in combination with atropine is injected to antagonize residual muscle relaxant effect. However, if myodynamia recovers fully, residual muscle relaxant effect will not be antagonized.

### Mechanical ventilation

During the interval between intubation and the change of body position, all patients are ventilated in pressure-controlled volume-guaranteed mode with 5cmH_2_O PEEP, with I:E fixed to 1:2, and respiratory rate adjusted in order to maintain blood carbon dioxide concentration within the range of 35-45 mmHg. Tidal volume is set to 8 ml/kg during two lung ventilation, both in supine and lateral decubitus positions. During one lung ventilation, tidal volume is set to 4-5 ml/kg.

After intubation in supine position, the tube lumen of the dependent lung is clamped; meanwhile, the operated lung is ventilated with pure oxygen until exhaled concentration of oxygen reaches 100%. Then, the tube lumen of the operated lung is clamped and FiO_2_ is immediately lowered to 0.4. In the meantime, fresh air flow is set to maximum to accelerate the decreasing of oxygen concentration in the ventilated lung. As soon as exhaled oxygen concentration reaches 0.4, both tube lumens are clamped, and patients are disconnected from the ventilator. Subsequently, patients are placed in lateral decubitus position followed by reconnection to the ventilator. The clamps on tube lumens are not removed until patients are reconnected to the ventilator. During the changing of body position, patients’ spine is kept in a straight line to avoid flexion and extension of the neck to prevent the displacement of the double lumen tube, which thereby reduces the time required to readjust the position of the tube. After confirming the position of the double lumen tube in lateral decubitus position, patients are ventilated according to their group assignment. Patients in both groups are ventilated with pure oxygen after adjusting the position of the double lumen tube in lateral decubitus position.

The moment after two lung ventilation is resumed, FiO_2_ is lowered to the largest extent (above or equal to 0.4) while not causing hypoxemia (SpO_2_ < 94%). During emergence, all patients are ventilated in synchronized intermittent mandatory ventilation (SIMV)-volume-guaranteed mode, with PEEP remaining the same with that of the intraoperative level, and FiO_2_ fixed till extubation unless hypoxemia occurs during emergence.

### Intervention

For patients in both groups, one lung ventilation is initiated after readjusting the position of the double lumen tube in lateral decubitus position, The lumen to the independent lung remain closed to air during the interval between intubation to pleural opening. It’s opened to air for 2 min after pleural opening, and subsequently connected with the apneic oxygen insufflation device, which delivers pure oxygen to the independent lung at a constant rate of 5L/min. The dependent lung is ventilated in pressure-controlled volume-guaranteed (PCV-VG) mode, with a tidal volume of 4-5 ml/kg, I:E set to 1:2, and respiratory rate adjusted to maintain end-expiratory carbon dioxide within the range of 30-40 mmHg.

**Fig. 2:**
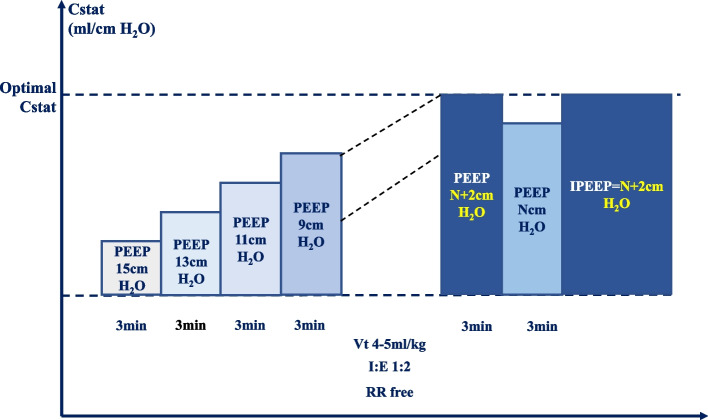
Individualized PEEP titrated to maximize Cstat. *RM* ventilator driven recruitment maneuver, *PEEP* positive end expiratory pressure, *iPEEP* individualized PEEP, *Cstat* static lung compliance

For patients in the study group, individualized PEEP titration is started right after the start of one lung ventilation. The initial PEEP is set to 15cmH_2_O. Subsequently PEEP is lowered at steps of 2cmH_2_O every 3 min. Before changing the level of PEEP, Cstat is calculated according to the formula (Cstat = VT/Pplat-PEEP). When Cstat ceases rising with the continuous lowering of PEEP but starts to drop, the previous PEEP level at which Cstat reaches its zenith is selected as the ideal PEEP (Fig. 2). Then PEEP is elevated to ideal PEEP and kept constant towards the end of the procedure.

**Fig. 3:**
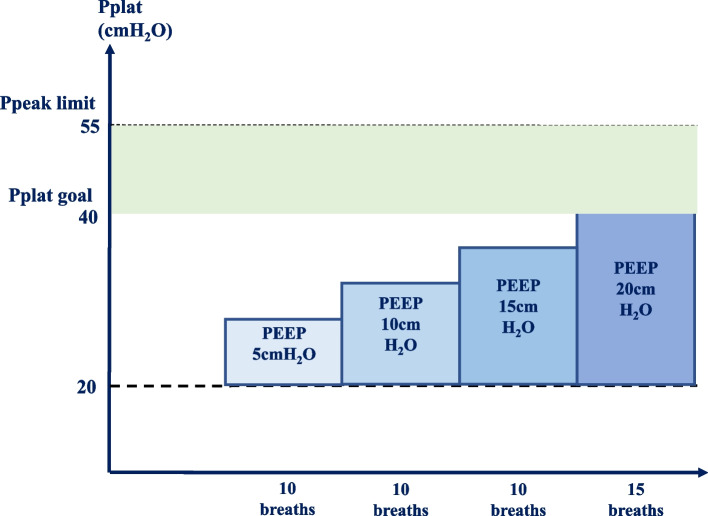
The ventilator-driven alveolar recruitment maneuver protocol. *P peak* Peak airway pressure. *Pplat* Plateau airway pressure. *PEEP* Positive end-expiratory pressure. *Vt* Tidal volume normalized for adjusted body weight. *I:E* Ratio between inspiratory and expiratory time. *RR* Respiratory rate

**Fig. 4:**
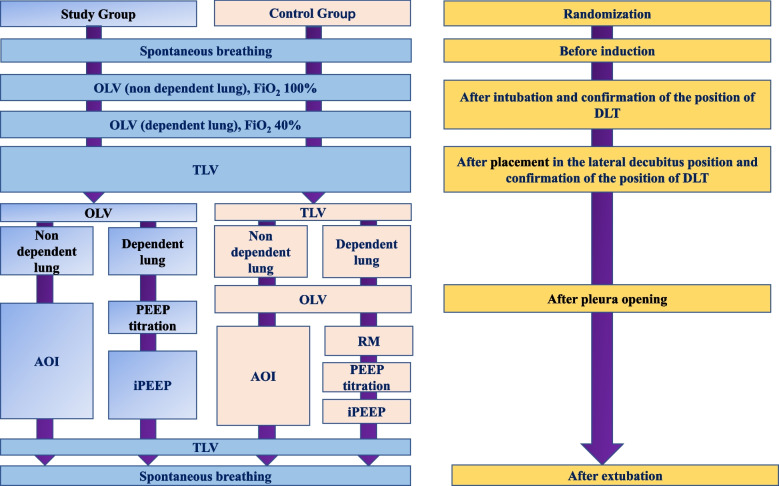
Intervention and perioperative management. *Group S* study group, *Group C* control group, *DLT* double lumen tube, *TLV* two lung ventilation, *AOI* apneic oxygen insufflation, *RM* ventilator driven recruitment maneuvers, *PEEP* positive end expiratory pressure, *iPEEP* individualized PEEP

For patients in the control group, PEEP is set to zero before pleural opening. Right after pleural opening, the dependent lung undergoes RM in pressure-controlled mode, driving pressure set to 20cmH_2_O, PEEP initially set to 5cmH_2_O and raised at steps of 5cmH_2_O and maintained for 10 breathing cycles for each step. When PEEP reaches 20cmH_2_O and plateau reaches 40 cmH_2_O, PEEP level is maintained for 15 breathing cycles towards the end of RM (Figs. 3 and 4), followed by iPEEP titration consistent with that of the study group.

### Protocol deviation

Anesthesiologists can deviate from the trial protocol whenever concerns over patients’ safety emerge. During one lung ventilation, if hypoxemia(SpO_2_ < 92%) persists after excluding displacement of double lumen tube, rescue recruitment maneuvers could be performed. Subsequently, two lung ventilation can be resumed temporarily in case hypoxemia is not corrected. Patients who deviate from the protocol will be excluded from the final analysis.

#### Strategies to improve adherence

The two main reasons for investigators to deviate from trial protocol are the occurrence of hemodynamic instability and refractory hypoxemia. So, during the pre-op visit, patients at risk for massive bleeding and hemodynamic instability should be identified. In addition, patients should be screened for risk factors of hypoxemia during one lung ventilation.Protocol amendments.

The primary investigator would notify all members of the research team, the ethics committee, and the surgeons, nurses and other anesthesiologists of our center who provide assistance to the implementation of the trial. After notification, a copy of the revised protocol would be added to the investigator site file.

#### Trial steering committee

The trial steering committee composes of colleagues of our department who supervise the progress of the trial and the integrity of collected data as well as anesthesiologists experienced in thoracic surgery anesthesia who provide valuable advice when unexpected adverse events arise. The trial steering committee meets every six months.

### Study endpoints

The primary endpoint of the study is oxygenation index: before induction (T0), after intubation (T1), after adjustment of the position of the double lumen tube in lateral decubitus position (T2), 5 min after PEEP titration and before pleura opening (study group) and 5 min after ventilating in lateral decubitus position (control group) (T3), after achieving satisfactory collapse of the independent lung (lung collapse index reaching 8 [13]) (study group) and 5 min after completing iPEEP titration and achieving satisfactory collapse of the independent lung (control group) (T4), before resuming two lung ventilation (T5).

Secondary endpoints are as follows:

1. Results of blood gas analysis.
2. Qs/Qt (intrapulmonary shunt) (calculated with results of blood gas analysis obtained at each time point).
3. Respiratory parameters (tidal volume (TV), respiratory rate (RR), plateau pressure (Pplat), static lung compliance (Cstat), driving pressure ($\Delta$ P)) recorded at the following time points: before and after RM, each step of iPEEP titration, before and after pleura opening, once every 15 min after pleura opening, before and after resuming two lung ventilation, before and after patients are turned into supine position at the end of surgery, before extubation.
4. Anesthesia-associated parameters: hemodynamic parameters, incidence as well as total duration of hypotension, time required for emergence from anesthesia, dose of anesthetics, incidence, and total duration of hypoxemia.
5. Hygienic indicators: incidence of transference to ICU, length of ICU stay, length of hospital stay, and adverse events.
6. PPCs. We define PPCs as follows:
  1. Respiratory infection: Two or more of the following for > 48 h: new cough/sputum production, physical findings compatible with pneumonia, fever > 38 $^\circ{\rm C}$, and new infiltrations on chest X-ray (CXR).
  2. Respiratory failure: Postoperative PaO_2_ < 8 kPa (60 mmHg) on room air, PaO_2_ / FiO_2_ (inspired fraction of oxygen) ratio < 40 kPa (300 mmHg), or arterial oxyhemoglobin saturation measured with pulse oximetry < 90% and requiring oxygen therapy.
  3. Pleural effusion: CXR with blunting of costophrenic angle, loss of sharp silhouette of the ipsilateral hemidiaphragm in upright position, displacement of adjacent anatomical structures, or (in supine position) hazy opacity in one hemithorax with preserved vascular shadows.
  4. Atelectasis: Lung opacification with mediastinal shift, hilum or hemidiaphragm shift towards the affected area, with compensatory hyperinflation in adjacent non-atelectatic lung.
  5. Bronchospasm: Newly detected expiratory wheeze treated with bronchodilators.
  6. Aspiration pneumonitis: Acute lung injury after inhalation of regurgitated gastric contents.
  7. Pneumonia: CXR with at least one of the following: infiltration, consolidation, cavitation; plus at least one of the following: fever > 38^。^C with no other cause, white cell count < 4 or > 12*10^9^ /L, > 70 yr of age with altered mental status with no other cause; plus at least two of the following: new purulent/changed sputum, increased secretions/suctioning, new/worse cough/dyspnea/tachypnoea, rales/bronchial breath sounds, worsening gas exchange.
  8. ARDS: Ventilated, bilateral infiltrates on CXR, PaO_2_:FIO_2_ < 300, minimal evidence of left atrial fluid overload within 7 days of surgery.
  9. Tracheobronchitis: Purulent sputum with normal chest radiograph, no intravenous (i.v.) antibiotics.
  10. Pulmonary oedema/Pulmonary congestion/hypostasis, acute oedema of lung, congestive heart failure, fluid overload.
  11. Exacerbation of pre-existing lung disease.

Patients conforming to one of the above conditions are deemed as positive for PPCs.

### Study visits and data collection

Patients are visited preoperatively, intraoperatively, and postoperatively on postoperative days 1 to 3 and at discharge (Table 1). We collect and record patients’ data at all the above-mentioned time points.

1. Preoperative indicators: age, gender, height, body weight, BMI, ASA status, hypertension, diabetes, chronic hepatic illnesses, gastric esophagus reflux illness, long term use of steroids, history of obstructive sleep apnea (receiving CPAP therapy or not), smoking (current smoker/ have quitted smoking for more than 8 weeks), forced expiratory volume in the first second /forced vital capacity(FEV1/FVC), FVC, preoperative blood gas analysis( PaO_2_, PaCO_2_), hemoglobin, albumin, alanine aminotransferase (ALT), aspartate aminotransferase (AST), urea, creatine.
2. Intraoperative indicators: Time required to achieve satisfactory lung collapse (lung collapse index reaching 8) from the moment of pleura opening, volume of blood loss, urine output, infusion volume (crystalloid fluid, colloid fluid), blood transfusion volume (heterotransfusion, autotransfusion), respiratory parameters (tidal volume, respiratory rate, plateau pressure, PEEP, driving pressure, Cstat, partial pressure of end-expiratory carbon dioxide, arterial blood oxygen saturation), hemodynamic parameters (blood pressure, heart rate, vasopressor), results of blood gas analysis, oxygenation index, Qs/Qt, duration of surgery, duration of anesthesia, duration of one lung ventilation, duration from placement in lateral decubitus position to pleura opening, side of surgery.
3. Postoperative indicators: incidence of PPCs, length of hospital stay, incidence of ICU transference, length of ICU stay.
4. Adverse events: adverse events will be monitored from the initiation of the trial intervention until discharge.

**Table 1:**
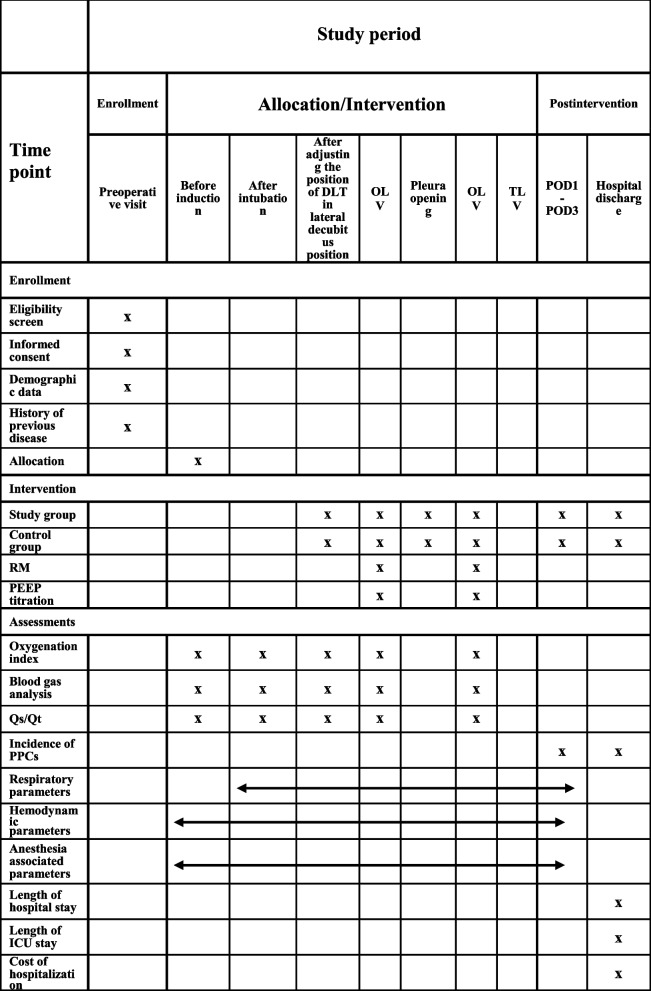
Standard Protocol Items: Time schedule of enrollment, interventions, and assessments

*DLT* double lumen tube, *OLV* one lung ventilation, *POD1* post-operative day 1, *POD3* post-operative day 3, *RM* Recruitment maneuver, *PEEP* Positive end-expiratory airway pressure, Qs/Qt fraction of intrapulmonary shunt, *PPCs* Postoperative pulmonary complications, *ICU* intensive care unit

### Sample size calculations

The primary endpoint of this study is oxygenation index measured at T4. According to literature [16], oxygenation index for patients inhaling pure oxygen and under well oxygenated conditions during TLV usually ranges between 350-400 mmHg and drops to 150-200 mmHg after patients are switched to OLV. Therefore, during OLV, changes in oxygenation index within the limit of 50 mmHg would be clinically insignificant. Thus, the cut-off value for inferiority is set to 50 mmHg. In other words, in our study, if oxygenation index measured at T4 of the study group is below that of the control group, but the difference between that of the two groups is within 50, then the ventilation strategy of the study group is not inferior to that of the control group in terms of improving intraoperative oxygenation. We set probability of type 1 error at α = 0.025, probability of type 2 error at β = 0.20, and power value at 0.80; a sample size of 38 is calculated for each group by using PASS 11.0 software. Considering a 10% removal and dropout rate, a final sample size of 42 is required for each group.

### Statistical analysis

The research team will cooperate with medical statisticians to analyze data after completing the trial. SPSS 20.0 software will be applied for data processing. Dropout or lost to follow up cases will be reported. Per-protocol analyses will be applied for all data. Under circumstances of missing values, multiple imputation shall be conducted. Kolmogorov–Smirnov analysis will be performed to discern normally distributed data. Normally distributed data will be presented as mean $\pm$ standard deviation (SD), while discrete data will be presented as median and interquartile range. Independent-samples T test will be adopted for univariate analysis of normally distributed data. Pearson correlation test will be used to test the linear relationship between two variables. Mann–Whitney U test will be applied for discrete data. Categorical variables will be expressed as relative risk ratio and assessed by Fisher’s exact test or chi-square tests, if applicable. 95% CI will be employed to express confidence level of all parameters. *P* value < 0.05 shall be defined as statistically significant.

#### Subgroup analysis

Patients will be divided into subgroups according to the side of surgery. Subgroup analysis will be conducted to further explore the effect of our trial intervention on intraoperative oxygenation. Post-hoc analysis will be applied for subgroup analysis.

## Discussion

Although atelectasis formation during anesthesia induction is difficult to avoid, it may have little influence on patients during one lung ventilation. Subjects of this trial are intubated in supine position before being switched to lateral decubitus position to receive one lung ventilation. In supine position, the pressure imposed by the lungs’own weight results in the reduction of trans-pulmonary pressure by 0.25cmH_2_O for every 1 cm on the ventral to dorsal axis. In consequence, for patients receiving mechanical ventilation in supine position, the dorsal parts of lungs are prone to collapse due to the reduction of trans-pulmonary pressure along the gravitation axis [17]. Tusman and colleagues discovered that by switching the body position of anesthetized children from supine to the left and subsequently right lateral decubitus position lasting 90 s each could recruit the collapsed alveoli in the dorsal parts of the lungs [18]. Mikulas and colleagues found that by tilting the less aerated lung of patients receiving mechanical ventilation for COVID-19-associated ARDS up 30˚ in combination with a decremental PEEP titration procedure could alleviate the degree of atelectasis [19].

When patients are placed in lateral decubitus position, the lower flank of the dependent lung is prone to collapse due to the impact of gravitational force. In our study, patients randomized to the group without RM receive one lung ventilation followed by immediate PEEP titration right after being placed in lateral decubitus position before the formation of atelectasis in the lower flank of the dependent lung. However, in most previous studies, patients received two lung ventilation without PEEP and inhaled pure oxygen after being placed in lateral decubitus position, and thus were prone to form atelectasis rapidly [1, 3–6]. Furthermore, in those studies, one lung ventilation was initiated after pleura opening and then patients received PEEP alone or received RM followed by iPEEP titration or a fixed level of PEEP. Hence, most previous researches compared the effects of PEEP applied alone or in combination with RM after the formation of atelectasis.

Recruitment maneuver could lead to temporal hemodynamic instability. Hence, the ideal recruitment maneuver eliminates atelectasis with the lowest effective peak inspiratory pressure and fewest numbers of breaths or shortest effective time [20]. Yet, evidence on the effective time and effective peak inspiratory pressure during recruitment maneuvers are still lacking. Although, the advantages of individualized PEEP has been proven by numerous studies, recruitment maneuvers applied in the majority of studies are unindividualized. Wu and collegues compared the effects of traditional recruitment maneuvers and ultrasound guided recruitment maneuvers on patients undergoing thoracic surgery [21].They discovered that, the incidence and severity of atelectasis are lower in patients receiving ultrasound guided recruitment maneuvers than those receiving conventional recruitment maneuvers. Therefore, unindividualized recruitment maneuvers may not be effective in recruiting all collapsed alveoli and thus has limited benefit. On the other hand, the pressure and time of unindividualized recruitment maneuvers may be excessive, aggrevating its potential harm.

Ideally, satisfactory collapse of the independent lung would be achieved to facilitate surgical procedure, while the atelectasis of the dependent lung is minimized to improve oxygenation and ventilation mechanics. Inhaling pure oxygen would facilitate the development of atelectasis, which plays a key role in the phase II collapse of the independent lung [22]. On the other hand, limiting the fraction of inspired oxygen could alleviate atelectasis [20].Thus, before placing the patient in lateral decubitus position, we lowered the fraction of inspired oxygen during ventilation of the dependent lung to avoid formation of atelectasis during adjustment of the position of the double lumen tube which necessities temporal disconnection from the ventilator. While the independent lung is ventilated with pure oxygen to facilitate the development of atelectasis.

The pressure required to open up collapsed alveoli is far higher than that needed to prevent atelectasis formation. So, if PEEP is incrementally titrated, atelectasis usually develops before PEEP reaches the level sufficient to prevent alveoli from collapsing, and thus limits the benefits of PEEP. Spadaro and colleagues discovered that during one lung ventilation, PEEP obtained via both decremental and incremental titration procedures could reduce intrapulmonary shunt, but only PEEP titrated decrementally succeeded in reducing driving pressure and improving oxygenation [24]. Chuimello and colleagues found that during a stepwise decremental PEEP titration procedure, keeping PEEP constant for 5 min at each step was adequate for arterial partial pressure of oxygen, oxygenation index, venous admixture and arterial blood oxyhemoglobin saturation to reach equilibrium [23]. In contrast, during a stepwise incremental PEEP titration procedure, keeping PEEP constant for more than 60 min was insufficient for the aforementioned parameters to reach equilibrium [24]. In the majority of studies, during a PEEP titration procedure, iPEEP was selected based on parameters associated with oxygenation and respiratory dynamics. In addition, in numerous studies, PEEP was kept constant for 40 s to 3 min after each step of a stepwise decremental or incremental PEEP titration procedure, which was far shorter than the time required for paremeters including oxygenation index to reach equilibrium [4, 5, 14, 23]. In conclusion, acquiring PEEP from an incremental PEEP titration procedure is inappropriate.

One lung ventilation could result in injury of both the independent and the dependent lung [25]. According to literature, during one lung ventilation, apneic oxygen insufflation to the independent lung at a fixed rate of 5L/min could retain a degree of its expansion, which not only contributes to mitigated inflammatory reaction but also alleviates the anoxic-reoxygenation injury acquired after the end of one lung ventilation [26]. Therefore, we will apply apneic oxygen insufflation to the independent lung at a constant rate of 5L/min to minimize the effects of its injury on the incidence of PPCs. Although surgery-related lung injury is beyond control, according to literature, detriment caused by ventilator-associated lung injury is far greater than that caused by surgical trauma [27]. Thus, surgery-related lung injury has no profound impact on the outcome of this study.

## Trial status

The protocol version is the first version (No. V1.0/2024.01.25). We will enroll the first participant of the trial on March 1, 2024, and complete recruitment on September 30, 2025.

## Supplementary Information

Supplementary Material 1.

## Data Availability

The datasets supporting the conclusions of this article are included within the article and its additional files.

## Abbreviations

ARDS: Acute respiratory distress syndrome
PPCs: Postoperative pulmonary complications
iPEEP: Individualized positive end-expiratory pressure
Cstat: Static lung compliance
FiO_2_: Fraction of inspired oxygen
RM: Recruitment maneuvers
V_T_: Tidal volume
CRF: Case Report Forms
BMI: Body mass index
CPAP: Continuous positive airway pressure
V/Q: Ventilation perfusion ratio
COPD: Chronic obstructive pulmonary disease
Pplat: Plateau airway pressure
Ppeak: Peak airway pressure
1:E: Ratio between inspiratory and expiratory time
PaCO_2_: Partial pressure of arterial carbon dioxide
PaO_2_: Partial pressure of arterial oxygen
ACEI: Angiotensin converting enzyme inhibitor
ARB: Angiotensin receptor blocker
DLT: Double lumen tube
OLV: One lung ventilation
Qs/Qt: Fraction of intrapulmonary shunt
TOF: Train of four
MAP: Mean arterial pressure
SIMV: Synchronized intermittent mandatory ventilation
PCV-VG: Pressure controlled volume guaranteed mode
AOI: Apneic oxygen insufflation
TLV: Two lung ventilation
FEV1: Forced expiratory volume in one second
FVC: Forced vital capacity
ALT: Alanine aminotransferase
AST: Aspartate aminotransferase
